# Natural Products-Based Drug Design against SARS-CoV-2 Mpro 3CLpro

**DOI:** 10.3390/ijms222111739

**Published:** 2021-10-29

**Authors:** Rai C. Silva, Humberto F. Freitas, Joaquín M. Campos, Njogu M. Kimani, Carlos H. T. P. Silva, Rosivaldo S. Borges, Samuel S. R. Pita, Cleydson B. R. Santos

**Affiliations:** 1 Graduate Program on Medicinal Chemistry and Molecular Modeling, Institute of Health Science, Federal University of Pará, Augusto Corrêa 01-Guamá, Belém 66075-110, PA, Brazil; tomich@fcfrp.usp.br (C.H.T.P.S.); lqfmed@gmail.com (R.S.B.); 2 Departamento de Química, Faculdade de Filosofia, Ciências e Letras de Ribeirão Preto, Universidade de São Paulo, Ribeirão Preto 14040-901, SP, Brazil; 3 Graduate Program on Pharmacy (PPGFAR-UFBA), Pharmacy College, Federal University of Bahia, Salvador 40170-115, BA, Brazil; humbarato@gmail.com; 4 Laboratory of Bioinformatics and Molecular Modeling (LaBiMM), Pharmacy College, Federal University of Bahia, Av. Barão de Jeremoabo, 147 Ondina, Salvador 40170-115, BA, Brazil; 5 Department of Pharmaceutical and Organic Chemistry, Faculty of Pharmacy, Campus of Cartuja, University of Granada, 18071 Granada, Spain; jmcampos@ugr.es; 6 Biosanitary Institute of Granada (ibs.GRANADA), University of Granada, 18071 Granada, Spain; 7 Department of Physical Sciences, University of Embu, Embu 6-60100, Kenya; njogu.mark@embuni.ac.ke; 8 School of Pharmaceutical Sciences of Ribeirao Preto, University of Sao Paulo, Ribeirao Preto 14040-903, SP, Brazil; 9 Laboratory of Modeling and Computational Chemistry, Department of Biological and Health Sciences, Federal University of Amapá, Macapá 68902-280, AP, Brazil

**Keywords:** COVID-19, 3CLpro, natural products, docking, molecular dynamics, druggability, ADMET properties

## Abstract

Coronavirus disease 2019 (COVID-19), caused by severe acute respiratory syndrome coronavirus 2 (SARS-CoV-2), has received global attention due to the serious threat it poses to public health. Since the outbreak in December 2019, millions of people have been affected and its rapid global spread has led to an upsurge in the search for treatment. To discover hit compounds that can be used alone or in combination with repositioned drugs, we first analyzed the pharmacokinetic and toxicological properties of natural products from Brazil’s semiarid region. After, we analyzed the site prediction and druggability of the SARS-CoV-2 main protease (Mpro), followed by docking and molecular dynamics simulation. The best SARS-CoV-2 Mpro complexes revealed that other sites were accessed, confirming that our approach could be employed as a suitable starting protocol for ligand prioritization, reinforcing the importance of catalytic cysteine-histidine residues and providing new structural data that could increase the antiviral development mainly against SARS-CoV-2. Here, we selected 10 molecules that could be in vitro assayed in response to COVID-19. Two compounds (b01 and b02) suggest a better potential for interaction with SARS-CoV-2 Mpro and could be further studied.

## 1. Introduction

Although coronaviruses (CoVs) have been known since 1940 [1], reports on human infections causing mild respiratory infections were first reported in the 1960s. In December 2019 [2,3,4,5,6], a new viral respiratory disease emerged from Wuhan, China, and rapidly spread globally. This virus was named SARS-CoV-2 because its genomic RNA is about 82% identical to the coronavirus that causes Severe Acute Respiratory Syndrome (SARS-CoV). The disease caused by SARS-CoV-2 is called COVID-19 [4]. On 11 March 2020, the World Health Organization (WHO) declared the COVID-19 outbreak a pandemic [3,4,5] and data indicate that the cumulative number of cases reported globally now exceeds 183 million, with fatalities of almost 4 million (https://www.who.int/emergencies/diseases/novel-coronavirus-2019, accessed on 9 June 2021).

Therefore, the rapid discovery of safe, effective, and broad-spectrum anti-COVID-19 drugs is urgent. Currently, there is no specific therapy for COVID-19 patients and drugs are limited [7], despite more than 80 clinical trials employing distinct drugs such as Chloroquine, Hydroxychloroquine, Arbidol, Remdesivir, Favipiravir, Lopinavir, Ritonavir, Oseltamivir, Methylprednisolone, and Bevacizumab [1,8]. Due to its relevance to viral replication, the Mpro enzyme, which plays a central role in mediating viral replication and transcription [4,7,9,10,11], was the main target investigated in this study and the compounds identified here will be evaluated in future assays.

Individuals infected with COVID-19 have flu-like symptoms with fever, headache, muscular soreness, cough, and dyspnea, and in a few cases, were reported vomiting and diarrhea. People with asymptomatic COVID-19 behaviors may have a longer incubation period, thus it is indicated that quick tracking of the infected individual is essential to limit person-to-person transmissions. The quarantine period of 14 days for travelers was considered recommendable. The reduction of social interaction by limiting activities at workplaces, education centers, and public functions were indicated to be more effective in preventing and reducing the rapid spread of infection [12,13,14,15].

Recent studies assume that supplementing one’s diet has been widely reported to improve the immunity, health, and microbiota of humans, which play a key role in the treatment of COVID-19, even in infected cases; healthy eating habits and symbiosis decrease the chances of acute inflammation and severe cases [16].

Natural products research and development (R&D) potentially plays a pivotal role in innovative drug discovery [17]. Medicinal plants have been employed by humans for thousands of years to treat illnesses, health disorders [18], and to identify some plants useful as antiviral drugs. Plants produce a high diversity of secondary metabolites with interesting biological activities and both their natural products and natural product-inspired agents have attracted significant attention because they have played an integral role in the treatment of many different conditions, such as viral infection, especially SARS-CoV-2 [18,19,20,21,22,23,24]. Carolacton, Homoharringtonine, Emetine, and Cepharanthine, as well as natural product-inspired small molecules (Ivermectin, GS-5734, EIDD-2801, and Ebselen), are potential anti-SARS-CoV-2 agents that have attracted significant attention due to their broad-spectrum antiviral activities [24].

A good starting point to find new antiviral natural products would be traditional medicinal plants. Brazil hosts about 20% of all the world’s biodiversity, being considered the country with the most endemic species worldwide [25]. The Natural Products Database of the Bahia Semi-Arid region (NatProDB, http://natprodb.uefs.br/, accessed on 7 May 2021) contains naturally occurring compounds with a wide chemical diversity. These compounds are yet to be fully explored for the discovery of bioactive molecules [26]. Thus, we searched for new molecular entities that could be potent inhibitors of SARS-CoV-2 Mpro in the NatProDB database.

Medicinal chemistry approaches have seen a great technological advancement over the years and have contributed immensely to the discovery of promising molecules. However, many compounds discovered by these techniques have shown unsatisfactory absorption, distribution, metabolism, excretion, and toxicity (ADMET) properties in in vivo tests [27].

**Figure 1:**
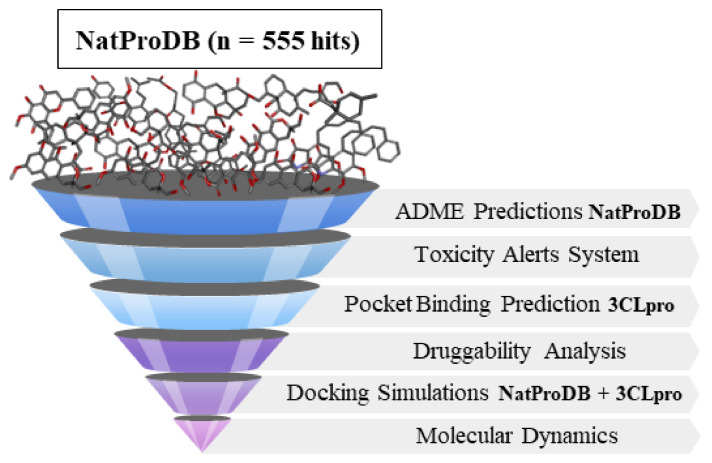
Virtual screening steps applied in this study to identify new compounds from NatProdB (555 hits) with potential antiviral activity against the SARS-CoV-2 Main protease, including: ADME property filters; toxicity alert filter for chemical groups; and docking simulations and molecular dynamics filters.

In this study, we propose to employ a structure-based drug design (SBDD) and ligand-based drug design (LBDD) approach against the main sites of the SARS-CoV-2 main protein (Mpro) through different computer-aided methods. We explore ADMET properties using two distinct tools, namely QikProp^TM^ and Derek^TM^, intending to reduce the selection of false-positive ligands and obtain hits that can be subjected to in vitro tests. We further evaluated the most likely corresponding bioactive conformations through tactics such as cavity detection, docking, and molecular dynamics (MD) simulations. All steps performed in this study were as described in Figure 1.

## 2. Results

In our previous work, we analyzed the NatProDB compound [28] interactions with a *Trypanosoma cruzi* enzyme. Here, we decided to analyze these data in further detail, employing additional distinct ADMET in silico methods: QikProp^TM^ and Derek^TM^ [29].

### 2.1. ADMET Analysis by QikProp^TM^

ADMET prediction was performed through the QikProp^TM^ module. This tool predicts some relevant properties (e.g., water/gas log Ps, log S, log BB, overall CNS activity, Caco-2, and MDCK cell permeabilities; log Khsa for human serum albumin-binding and log IC_50_ for HERG K+-channel blockage) based on the full 3D molecular structure over twenty physical descriptors [30].

First, we collected 555 molecules from the NatProDB using the filter #star to calculate the number of properties that were outside the range by comparing a particular molecule’s properties with those of the 95% of known drugs via QikProp^TM^ [30,31,32]. Although it is acceptable to have at least five properties per hit outside the range, in this study, all hits with #star values of ≥1 were eliminated following the E64 (positive control), yielding 376 compounds. Subsequently, we evaluated the predicted activity on the central nervous system (CNS), which ranged from −2 (inactive) to +2 (active) [32,33,34]. Only compounds with values of <0 were kept (203 hits) considering that the antiviral action mechanism proposed in this study does not necessarily require an effect on the CNS.

Next, the human oral absorption properties [35] were predicted for the remaining compounds using the values of a percentage ranging from >80% (high and desirable) to ≤25% (low absorption) as standard criterion [32,33]. Aiming to search for good absorption potential, molecules with values of ≥60% were maintained since E-64 presented 14.41%, obtaining 201 candidates.

To assess the apparent permeability on the Caco-2 cell membrane, the Boehringer-Ingelheim scale (nm/s) was employed. Compounds with low permeability presented <5 nm/s and those with high permeability showed values of >500 nm/s [30,31,32]. In this process, only molecules with values above 100 nm/s were considered and yielded in the 153 molecules with majorly pharmacokinetic desirable properties for the top 10 hits (Supplementary Table S1).

### 2.2. Toxicological Analysis by Derek Nexus^TM^

For the toxicity analysis predictions, we sought to identify and eliminate molecules that exhibited any toxicity alert (carcinogenicity, mutagenicity, genotoxicity, skin sensitization, teratogenicity, nephrotoxicity, hepatotoxicity, irritation, respiratory sensitization, and reproductive effects) [29] for specific chemical groups in the early stages of the process for developing prototype drugs, making it possible to optimize costs and time during research. Thus, the evaluation of chemical groups of the remaining 153 NatProDB compounds resulted in 47 candidates with low toxicological potential and adequate pharmacokinetic profiles for an oral route, i.e., good intestinal absorption, high cellular permeability, and low excretion, thereby promising antiviral candidate drugs.

The polar molecular surface (PSA) area is a property based on polar atom surface that was shown to correlate with passive molecular transport through membranes and allow for the prediction of the transport properties of drugs [36,37]. Its desirable value ranges from 7.0 to 200.0 Å^2^ [30,31,32]. Molecule 6 had the highest PSA index of 107.35 Å^2^, while compound 10 had the lowest value at 46.80 Å^2^. This indicates a good permeability of the selected molecules through the lipid membrane. It is noteworthy that a PSA value of <75 Å^2^ has been associated with an increased risk of adverse effects when combined with high lipophilicity (logP greater than 4) and only one matched that situation, which was excluded [36].

A drug bound to a protein is inactive and it is only the free drug that can bind with a receptor, providing pharmacological action [38]. The free drug concentration is affected by binding on human serum albumin (HSA), therefore this pharmacokinetic property was also predicted for the compounds [39,40]. This was evaluated by its logarithmic constant and has a recommended range of −1.5 to 1.5 [30,32,41]. Values outside the standard range may indicate non-enzymatic glycosylation of HSA interference on drug-binding potency, and may influence the distribution and excretion of molecules affecting the duration and intensity of the biologic effects [40,42,43]. All molecules selected showed satisfactory values that were better than the E64, which was the smallest in the series, specifically logKHSA = −1.31 (Table S1).

Finally, the hERG encodes the inward rectifying voltage-gated potassium channel in the heart (IKr), which is associated with cardiac repolarization. Inhibition of the hERG current induces QT interval prolongation and could represent fatal ventricular arrhythmia called Torsade de Pointes [44]. In fact, recent studies pointed out that some medications such as Hydroxychloroquine and azithromycin have facilitated the risk of fatal arrhythmia in patients with COVID-19 infection [45,46]. In our study, molecules that presented QPlogHERG values below −5 (2 = −5.14; 4 = −5.53 and 5 = −6.48) required greater attention and further in vitro analysis should be done to validate their pharmacological safety in the treatment against COVID-19.

### 2.3. Site Prediction and Druggability Analysis of SARS-CoV-2 Mpro

**Figure 2:**
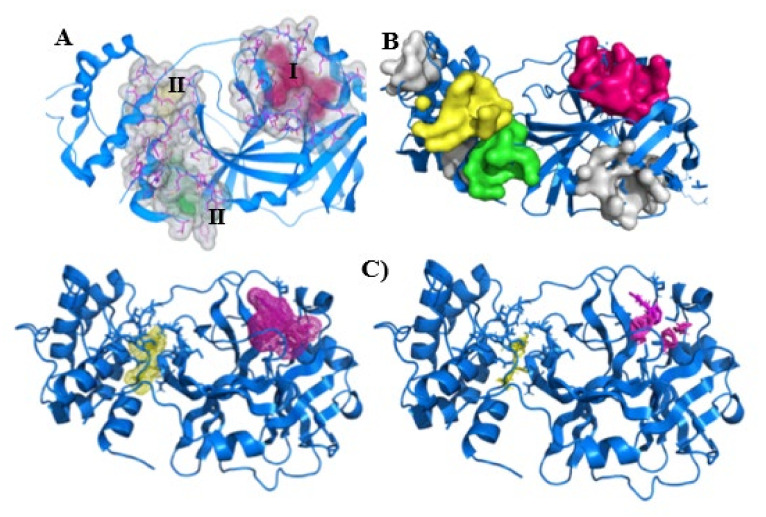
Three-dimensional structure of the SARS-CoV-2 main protease (SARS-CoV-2 Mpro, PDB ID: 5R82, blue cartoon). (**A**) Binding sites (surface and pink sticks) found by SeeSAR version 9.2 (BioSolveIT GmbH) in the active site (I) are colored in pink (25 residues), at site II (19 residues) in yellow, and at site III (15 residues) in green. This image was generated by the SeeSAR program. (**B**) Main pockets (color surfaces) predicted by PockDrug server [47]. The active site (I) is colored in pink (volume = 1892 Å^3^, druggability score = 0.63 ± 0.09), at site II (volume = 643 Å^3^, druggability score = 0.35 ± 0.08) in yellow, and at site III (volume = 760 Å^3^, druggability score = 0.33 ± 0.03) in green. The remaining gray sites presented a druggability score of 0.55 ± 0.10 (upper), 0.98 ± 0.01 (middle), and 0.61 ± 0.04 (lower). (**C**) Main hot spots (mesh and sticks) calculated by FTMap server [49]. The active site is colored in pink and was classified as druggable (≥16 probes) [54], the second site (yellow) is borderline (13 ≤ probes < 16), and pink and yellow sticks show the location of the probe. The B and C images were generated by educational Pymol 2.4.1 [55].

We noted that all programs correctly identified the SARS-CoV-2 Mpro active site (Figure 2A–C) and additionally indicated that this site was druggable, e.g., with a druggability probability of >0.5 in PockDrug [47] as well as a strong primary hot spot with ≥16 probes clusters and hot spots connected with a center-to-center distance of <8 Å in FTMap [48,49]. These agree with all experimental SARS-CoV-2 Mpro structures [4,7,9,50,51], where distinct ligands were tested and crystallized.

After the protein preparation step, pocket predictions and the druggability analysis of the SARS-CoV-2 Mpro were done using three different methods: Difference of Gaussian (DoG) [52,53], PockDrug [47], and FTMap [49]. The main pockets determined in this protein are shown below (Figure 1)

Other sites were also signed as druggable in our analysis: SeeSAR (II and III—Figure 2A); PockDrug (green, yellow, and gray sites—Figure 2B); and FTMap (yellow—Figure 2C). Since two SARS-CoV-2 Mpro sites were located in the same locations and were specified by distinct methods (II—yellow surface and yellow mesh; III—green surface and yellow mesh, Figure 2), we hypothesized that these two regions could be explored in further studies.

The probes posed by FTMap revealed that the druggable site (magenta sticks, Figure 2C) accommodates polar acyclic compounds (ethanol, dimethylformamide, and acetamide) and some rings (benzene, benzaldehyde, and cyclohexane), and the borderline site (yellow sticks, Figure 2C) lodges ethanol, methanamine, benzene, and cyclohexane. This information is helpful for fragment-based drug design. In another further step, gray sites predicted by PockDrug (Figure 2B), which had higher druggability scores, could also be explored.

### 2.4. Molecular Docking Simulations

The docking calculations were done by SeeSAR version 9.2 (BioSolveIT GmbH) and focused on the active site of SARS-CoV-2 Mpro to better sample the interaction energy (ΔG) between NatProDB ligands and this enzyme. The hydrogen bond and dehydration (HYDE) energy scoring function in SeeSAR provides a range of affinities, spanning an upper and lower limit. 

We, therefore, used this descending range as an affinity parameter to compare distinct molecules and their interactions on the Mpro active site. For the sake of clarity, we only show the 10 best-docked compounds with higher estimated affinities (EA and µM) in Table 1 instead of 46 NatProDB and E-64 molecules as previously filtered from the ADMET analysis (see preceding discussions).

SeeSAR version 9.2 (BioSolveIT GmbH) analyzes the estimated affinities’ (EA) energies and the best poses selection was based on both the HYDE scoring function [61,62,63] and torsional analysis [64]. The docked molecules at the SARS-CoV-2 Mpro active site were ordered by their EA on SARS-CoV-2 Mpro-NatProDB complexes and the “top 10” were selected for further analysis.

In comparing the docking results presented in Table 1, we noted that NatProDB compounds’ energy ranked in the “top 10” were better ranked than the Mpro crystallographic ligand (RZS, 255th better energy). These results indicate a good interaction between NatProDB and SARS-CoV-2 Mpro residues, mainly on the active site. Additionally, the “top 10” NatProDB compounds presented better energy when compared to a positive control (E-64), which was a well-known cysteine protease inhibitor (562th better energy, Table 1) [65,66]. 

The best-scored poses were selected by visual inspection and, since they interacted better with Mpro active site residues, we decided to compare their interactions by analyzing each complex (Mpro- RZS, E-64, and NatProDB), aiming to find some structural information that could help regarding the drug discovery against COVID-19 (Figures S1–S4).

These hits occupied the active site in a varied manner and interacted with Mpro through hydrogen bonds with catalytic residues (His41 and Cys145), as well as made additional hydrophobic contacts as shown from the docking results (Figures S1–S4).

The main interactions were described by the Protein–Ligand Interaction Profiler server (https://plip-tool.biotec.tu-dresden.de/plip-web/plip/index, accessed on 27 May 2021) [67] at each pose (Table S2), where we noted that the two better-docked NatProDB compounds (b01 and b02) presented better contacts than the crystallographic ligand (RZS).

### 2.5. Molecular Dynamics (MD) Simulations

To understand the behavior of SARS-CoV-2 Mpro complexes, we analyzed them through molecular dynamics (MD) simulations. These data allowed us to analyze the time influence on these interactions’ patterns. First, we analyzed the stability of our eight systems: apo and complexes: holo (PDB ID: 5R82), as well as E-64 and the five better-ranked NatProDB compounds (Table S3). From our root-mean square deviation (RMSD) data, we noted that all the systems equilibrated after 40 ns (Figure S5A). Then, we defined our productive phase into time intervals from 40 to 100 ns for all the simulations (Figure S5B,C).

**Figure 3:**
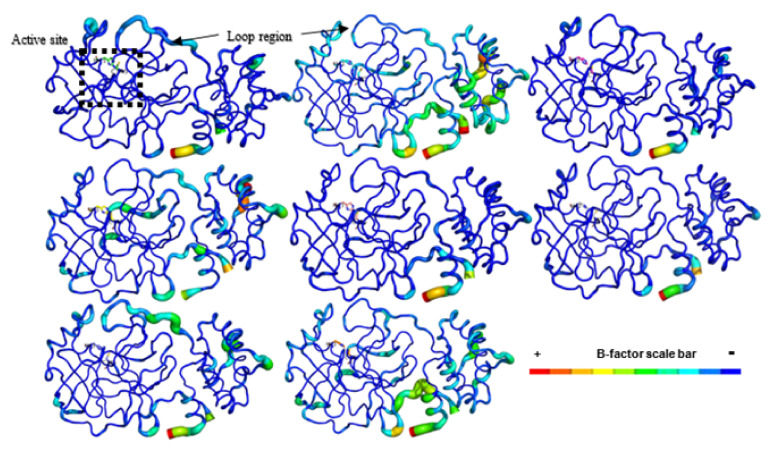
SARS-CoV-2 Mpro structures are shown in B-factor putty mode in PyMOL. The highest B-factor in each structure is colored in red and the lowest in dark blue, as indicated by the B-factor scale bar. The thickness of the protein backbone also is proportional to the B-factors. The catalytic residues (His41 and Cys145) are displayed as sticks. This image was generated by educational Pymol 2.4.1 [64].

In comparing the Mpro structure fluctuations (3D RMSF) within all the complexes, we distinguished their overall protein-folding stability, which was mainly at the active site represented by catalytic residues (His41 and Cys145, Figure 3). These data correlated with the Mpro crystallographic structure, where active site residues were well solved in the electron density maps from 2.1 Å resolution [7].

Additionally, we analyzed their secondary structure stability by the DSSP 3.1.4 module [68,69,70], which was installed on GROMACS 5.1.4 [71,72,73,74,75,76]. All Mpro complexes maintained their secondary structure stability during our simulation time (Figure S6). Some regions presented higher fluctuations in Mpro structure, mostly at some loops and at both N and C-terminals (Figure 3). The SARS-CoV-2 Mpro crystal had a long loop region of domain II (residues 185–200) connecting to domain III and this region is highly variable as emphasized by the superposition of 12 Mpro crystal structures [7].

Our MD data revealed that these loops on domain II fluctuated higher than other secondary protein structures, corroborating with this experimental study. Previous results also cited the interface region among Mpro protomers composed by domain II residues as unstable [4,7,9] and these data were also visualized in our simulations (Figure 3).

Many studies described Mpro subsites expanding from S6 to S1 with catalytic sites located on S1 [4,7,9,51]. Based on these results, we analyzed the interaction pattern on SARS-CoV-2 Mpro of NatProDB derivatives compared with the crystallographic ligand (RZS) and E-64. Additionally, we evaluated the hydrogen bond pattern, through the GROMACS H-bond module [77] and HbMap2Grace program [78], and the molecular surface area, by the SurfinMD program [79]. The molecular dynamics (MD) data for the hydrogen bond (H-bond) analysis showed that NatProDB compounds exhibited more interactions than RZS, and b04 and b05 were similar to E-64, which has the highest H-bond number (Table S3). Since these interaction patterns could favor their inhibitory behavior, we analyzed them in detail. Initially, we defined and calculated the hydrogen bond capability for our ligands (Table S3), (Hbondcapac.), during the MD simulation. This measure could aid us in quantifying how “tightly” the ligands interacted with Mpro since Hbondcapac. ≥ 1 indicates that each ligand’s hydrogen bond atoms (donors and acceptors) interacted with protein residues.

**Figure 4:**
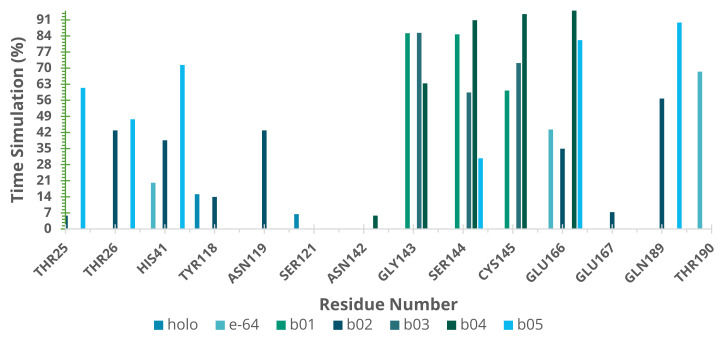
Hydrogen bond stability in SARS-CoV-2 Mpro complexes as calculated by HbMap2Grace after 100 ns of molecular dynamics simulation.

Our results from the MD productive phase correlates well with the estimated binding energy obtained before from the docking results (Table 1), i.e., the ligands with the best docking energies presented higher Hbondcapac., which revealed to us that the hydrogen bond is a favorable interaction in developing Mpro inhibitors. Additionally, we noted that our ligand accessed distinct sub-sites on SARS-CoV-2 Mpro, interacting beyond the catalytic ones—S1 (Figure 4).

The H-bond pattern presented interactions with His41 and Cys145 (catalytic residues); Thr25 and 26 from S1′; Gly143 and Ser144 from the canonical oxyanion role on S1 [7]; Glu166 from S1 and Gln189; and Thr190 from S4 [4,7,9,51]. Since the H-bond is considered as the “driving force” for Mpro inhibition, we could map and analyze them through our MD simulations.

**Figure 5:**
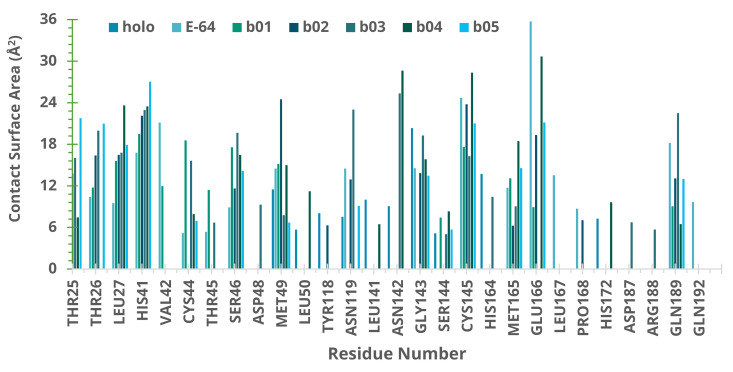
Surface molecular area (Å2) of SARS-CoV-2 Mpro complexes as calculated by SurfinMD after 100 ns of molecular dynamics simulation.

We also calculated the atomic contacts involving SARS-CoV-2 Mpro and NatProDB compounds (Figure 5). The contact surface area revealed additional interactions with apolar residues on the same sites described before [4,7,9]: S1′ (Thr25, 26, Leu 27, and Cys145), S2 (His41, Thr45, Ser46, Asp48, and Met49), S4 (Met165, Glu166, Leu 167, and Gln189), and S5 (Pro168).

Another measure to evaluate Mpro interaction with NatProDB ligands concerns their active site occupancy. Ideally, it is expected that potent inhibitors should present reversible action mechanisms and tight binding [80]. Since Mpro active sites have a well-defined area (335.9 Å^2^) and volume (364.1 Å^3^) [81], we compared these results with our MD data. The Fpocket program [82,83] was employed to calculate the volume variation throughout the simulation. We noted that the active site volume oscillates from 461.8 Å^3^ of E-64 complexes to 262.9 Å^3^ for **b04** (Table S3), which agreed well with previous data [4,50,81] and with both the molecular area and volume of NatProDB ligands (Table 1). These results could emphasize that SARS-CoV-2 Mpro and NatProDB ligands show induced-fit mechanisms, correlating with their dynamics and molecular interactions at the active site.

**Figure 6:**
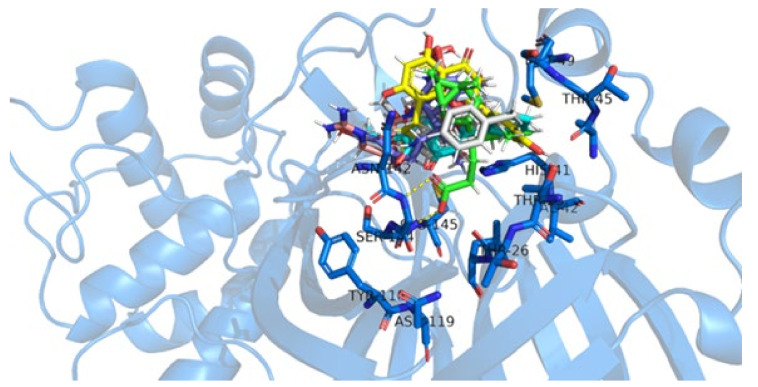
SARS-CoV-2 Mpro main residues that interacted with the best-docked NatProDB compounds throughout the productive phase of the molecular dynamics simulation. Mpro is shown as a cartoon (blue) with its main interacting residues labeled (blue sticks); b01 is shown in green sticks; b02 in cyan; RZS in magenta; and E-64 in orange. Polar interactions are depicted as yellow dashed lines. This image was generated by educational Pymol 2.4.1 [64].

Based on the interaction results of complexes after MD simulations, we assumed that new Mpro sites were pointed to: (i) V42, C44, T45 and S46, and both near and on (ii) Y118 and N119 (Table S3). The former residues made hydrophobic interactions with alpha-ketoamide inhibitors as described before [4], including FDA-approved drugs, medicinal plant compounds [81], and ZINC-15-docked compounds [84]. Our results (Figure 6) reinforce the importance of these non-polar interactions and opens up a new region (together with latter residues) for drug discovery development, aiming to search for new Mpro inhibitors.

**Figure 7:**
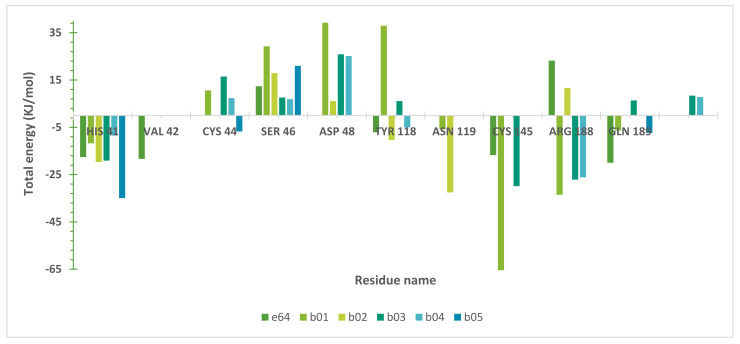
Residue contributions to the binding energy of SARS-CoV-2 Mpro complexes. The main residues with energy interactions (ΔEbinding > ±5 kJ/mol) are highlighted. For Mpro sub-site definitions, see the text.

Additionally, we calculated the binding free energy of all Mpro complexes through MM-PBSA methods [85,86]. The binding energy (ΔEbinding) calculated by the solvent accessible surface area showed that all compounds interacted favorably with Mpro (Table S3). Since these values were directly correlated to interacting protein residues, we decided to discriminate how amino acids presented better contacts with ligands. These residue decomposition energy analyses selected residues near the ligand (<5 Å) during the MD simulation and also those that participated actively in complex stabilization (ΔEbinding > ±5 kJ/mol), as shown in Figure 7.

We noted that ligand interactions with catalytic residues (His41 and Cys145) are highly favorable (negative values), as expected for reversible inhibitors. This behavior remained for two residues of the new binding site (Arg188 and Gln189), as first described in this work. Other residues (Val42, Cys44, Ser46, and Glu48) did not interact favorably with the ligands (positive values—Figure 6). This could be related to their position on the Mpro site, i.e., their side chains were pointed outwards instead of towards the active site cavity [4,9].

## 3. Discussion

Natural products represent an important source in discovering new and effective therapies to treat patients with COVID-19. Plant extracts are used around the planet as natural immune stimulants and anti-infective agents [87]. A good starting point to find new antiviral natural products would be traditional medicinal plants, such as those from Asia, Africa, or America that have been employed to treat infections. Progressive understanding of the development of efficient antiviral drugs and their bioactivity mechanisms has encouraged the exploration of natural products as an important way to indicate effective treatments for patients with COVID-19. Computational studies obtain and provide comparative approaches in a fast and economical way [88,89].

Carolacton is an antibacterial macrolide keto-carboxylic acid synthetized by the myxobacterium *Sorangium cellulosum* [90] and is reported as an inhibitor of Methylenetetrahydrofolate dehydrogenase 1 (MTHFD1), which is a potential target for developing anti-SARS-CoV-2 agents. The Carolacton strongly inhibited SARS-CoV-2 replication in Vero cells at a half-maximal inhibitory concentration (IC50) and exhibited a moderate cytotoxicity (CC50) profile [24,91].

Tetrahydroisoquinoline alkaloid emetine, a known natural product that was isolated from the plant *Psychotria ipecacuanha* [92], showed potential cardiotoxicity and acted as a protein synthesis inhibitor [93]; notably, this natural product has very recently been recognized as a promising antiviral drug with in vitro activity against MHV-A59 [94], severe acute respiratory syndrome coronavirus (SARS-CoV), and Middle East respiratory syndrome coronavirus (MERS-CoV) [95]. Recently, researchers performed a virtual screening against the SARS-CoV-2 Mpro binding site using the library of Marine Natural Products. Through many comparative prediction approaches, they selected seventeen most promising compounds in terms of being potential SARS-CoV-2 inhibitors [96].

The Natural Products Database of the Bahia Semi-Arid region (NatProDB, http://natprodb.uefs.br/, accessed on 7 May 2021) contains naturally occurring compounds with a wide chemical diversity. NatProDB phytocompounds were explored for their antiviral potential using a ligand and structure-based drug design approach against the main sites on the SARS-CoV-2 main protein (Mpro) through different computer-aided methods. Many bioactive compounds do not progress to clinical phase trials due to undesirable pharmacokinetic properties identified in advanced stages of the drug design, representing a significant loss of money, equipment, and time. Thus, it is essential to understand the pharmacokinetic profile of potential drug candidates at an early stage of research and development [41,97,98].

Compound b05 had the highest PSA index (107.35 Å^2^), listed in Table S1, compared to both the b08 and E-64 control (46.80 Å^2^), which were the lowest values of the series. Such evidence suggests good permeability of the molecule in a cellular lipid membrane [35]. A low PSA (PSA < 75 Å^2^) has been associated with an increased risk of adverse events due to non-specific toxicity, particularly when combined with high lipophilicity (log P > 4) [36]. A high PSA value has been associated with poor membrane permeability and, in particular, with low blood–brain barrier penetration, which denotes a specific structure that protects the central nervous system (CNS) [99]. For good oral bioavailability, an upper limit of 140 Å^2^ has been suggested, particularly when associated with a large number of rotatable bond surfaces belonging to polar atoms [100].

Cerebrovascular diseases are among the comorbidities of patients with confirmed COVID-19 who develop severe respiratory complications [101]. The intense systemic inflammatory response linked to viral infection can lead to blood–brain barrier (BBB) breakdown. This in turn can allow peripheral cytokines to gain access to the CNS, where they may trigger or exacerbate neuroinflammation, leading to encephalitis [102,103]. Recently, studies estimated the incidence of neurological syndromes to be about 37% [104,105,106]. In Table S1, it is possible to visualize that the b01, b04, b05, and b08 compounds presented a CNS activity value of −1, suggesting that they can be more active than the E-64 control (−2). For a drug with biological activity in the CNS region, a value higher than −2 is required [107,108].

The angiotensin-converting enzyme (ACE2) is expressed in Caco-2, Calu-3, and Vero-6 cells on the apical membrane domains [109,110]. A study investigated a library of thousands of marketed drugs and prototypes to evaluate the inhibition of virus-induced cytotoxicity using Caco-2 cell lines from human epithelial colorectal adenocarcinoma and SARS-CoV-2 obtained from a patient originally infected with the virus in Wuhan, China. Thus, 19 compounds with IC50 < 1 μM were identified, including the Remdesivir [111], exhibiting the same inhibition of SARS-CoV-2 in Caco-2 and Vero-6 cells (IC_50_ 0.77 μM), and surprisingly, promising potency (IC_50_ 0.07 μM) in SARS-CoV and MERS [112]. In our analysis, only hits which presented high Caco-2 values considered. These included compounds b02 (759.23 nm/s) and b04 (1101.54 nm/s). The others exhibited values between 5 nm/s and 500 nm/s. All hits had better values than the E-64 control (1.52 nm/s), considering the low apparent permeability on the Caco-2 cell membrane. This suggests that the compounds may be transported across the host cellular membrane or may block the virus–host membrane interaction that is critical for the progression of COVID-19 infection. 

To achieve better permeability, the drug should have a moderate logP value (−2 to 6.5). In this range, there is likely to be a good balance between permeability and solubility [113,114]. LogP values in Table S1 show that three bioactive compounds had higher affinity for the organic phase, characterized as more hydrophobic, in the descending order of b01, b04, and b02. All bioactive compounds presented optimum logP values within the range, favoring good permeability and solubility for drugs, with molecule b03 (0.15) being the most hydrophilic. In our analysis, logP values were directly related to the values of human oral absorption (HOA%). The E-64 control presented a value (−3.93) much lower than necessary for what is considered adequate for logarithmic partition coefficients (n-octanol and water phases).

The site prediction and druggability analysis of SARS-CoV-2 Mpro was identified by the probes posed by FTMap and revealed the druggable site (magenta sticks, Figure 2C) which accommodates polar acyclic compounds (ethanol, dimethylformamide, and acetamide) and some rings (benzene, benzaldehyde, and cyclohexane), as well as the borderline site (yellow sticks, Figure 2C) which accommodates ethanol, methanamine, benzene, and cyclohexane. This information is helpful for fragment-based drug design. In a further step, gray sites predicted by PockDrug (Figure 2B), which had higher druggability scores, could also be explored. These agreed with all experimental SARS-CoV-2 Mpro structures, where distinct ligands were tested and crystallized [4,7,9,50,51].

The initial molecular docking analysis revealed that all the selected NatProDB phytocompounds against the SARS CoV-2 3CLpro occupied the active site in a varied manner, interacted with Mpro through hydrogen bonds with catalytic residues (His41 and Cys145), and made additional hydrophobic contacts as shown from the docking results in Figures S1–S4. The main interactions were described by the Protein–Ligand Interaction Profiler server (https://plip-tool.biotec.tu-dresden.de/plip-web/plip/index) [67] at each pose (Table S2), where we noted the two best-docked compounds, thus only the interactions of both with 3CLpro were analyzed in detail and showed satisfactory intervals between the lower and upper limits of the estimated affinities b01 (3.29–326.4 µM) and b02 (4.42–439.6 µM). In addition, both had better contacts than the crystallographic ligand (RZS), as depicted in Figures S1–S4.

The atomic clashes between the protein and docked ligand atoms (inter-clash type), as predicted by SeeSAR9.2 (BioSolveIT, Sankt Augustin, Germany, 2018, www.biosolveit.de/SeeSAR, accessed on 25 July 2021) were classified as + (no clashes), 0 (few clashes), and (many clashes) Table 1. All hits exhibited few or no clashes, including the RZS and E-64 controls. This suggests the appropriateness of the docked conformations generated from the docking tool and that these docked conformations can have equivalent intermolecular electron density overlaps similar to the experimental SARS-CoV-2 3CLpro data. Further validation of these conformations in MD simulations showed that the b01, b02, and RZS conformations show a stable interaction with SARS-CoV-2 3CLpro. Our results from the MD productive phase correlated well with the estimated binding energy obtained before from the docking results (Table 1). These ligands with the best docking energies presented higher Hbondcapac., which revealed to us that the hydrogen bond is a favorable interaction in developing Mpro inhibitors.

Ideally, it is expected that potent inhibitors should present reversible action mechanisms and tight binding [80]. Since the Mpro active site has a well-defined area (335.9 Å^2^) and volume (364.1 Å^3^) [81], we compared these results with our MD data. The Fpocket program [82,83] was employed to calculate the volume variation throughout the simulation. We noted that the active site volume oscillates from 461.8 Å^3^ of E-64 complexes to 262.9 Å^3^ for b04 (Table S3), which agreed well with previous data [4,50,81] and with both the molecular area and volume of NatProDB ligands (Table 1). These results could emphasize that SARS-CoV-2 Mpro and NatProDB ligands show induced-fit mechanisms, correlating with their dynamics and molecular interactions at the active site.

The H-bond pattern presented interactions with His41 and Cys145 (catalytic residues); Thr25 and 26 from S1′; Gly143 and Ser144 from the canonical oxyanion role on S1 [7]; Glu166 from S1 and Gln189; and Thr190 from S4 [4,7,9,51]. Hence, it can be concluded that the docking procedure generated correct poses for the tested compounds. Finally, binding free energy calculations and the decomposition analysis showed a good binding affinity of b01 and b02. These ligands interact with the key 3CLpro residues, including a strong interaction with the Cys145–His41 catalytic dyad (Figures S3 and S4), which is crucial for the 3CLpro function [115,116,117,118]. 

Altogether, the present investigation provides a comprehensive overview of Bahia Semi-Arid region (NatProDB) compounds and shows their potential against SARS-CoV-2 3CLpro. The in silico analysis elucidated two potential candidates, namely b01 and b02 as potential 3CLpro inhibitors. However, both are subject to some uncertainty and additional in vitro and in vivo studies are essential to validate their efficacy as good drug candidates for the inhibition of 3CLpro activity [119].

## 4. Materials and Methods

Molecular modelling calculations, estimations, and visualizations of SARS-CoV-2 Mpro binding affinities were carried out using the SeeSAR version 9.2 software package (BioSolveIT GmbH) with the HYDE visual affinity assessment [61,62,63] carried on a personal computer with 4 logical processors of an Intel^®^ Core TM i5-6200U and 2.3 GHz processor using the Windows 10 Home operational system.

### 4.1. SARS-CoV-2 Mpro Protein Preparation

The high-resolution X-ray crystallographic structure of SARS-CoV-2 Mpro (1.31 Å) was obtained from the Protein Data Bank (PDB ID: 5R82, [120]) and was imported into SeeSAR version 9.2 (BioSolveIT, Sankt Augustin, Germany, 2018, www.biosolveit.de/SeeSAR, accessed on 25 July 2021). The structures were then prepared using the Protein Editor tool as implemented in the BioSolveIT Suite. Briefly, the raw PDB structure was processed by automatically assigning bond orders, adding hydrogens, adding missing side-chains, creating possible disulfide bridges, deleting waters, and generating hetero protonation states at pH 7.4. Residues with alternate positions were locked in the conformations with the highest average occupancy. Small ligand (6-(ethylamino) pyridine-3-carbonitrile-RZS) and dimethyl sulfoxide (DMS) originating from the crystallization buffer were removed. The hydrogen-bonding networks were optimized automatically by sampling water orientations and optimizations of hydroxyls, Asn, Gln, and His residue states using the Protein Editor.

### 4.2. Site Prediction and Druggability Analysis of SARS-CoV-2 Mpro

#### 4.2.1. SeeSAR

The protein structures prepared as described above were exported as PDB files and inserted on the “Binding Site” tool using BioSolveIT Suite. The site predictions and druggability analysis were determined by applying the Difference of Gaussian (DoG) site pocket finder [52,53,121]. DoGSite provides the functionality to detect potential binding pockets and sub-pockets of a protein of interest. Subsequently, it analyzes the geometric and physico-chemical properties of these pockets and estimates druggability with the aid of a support vector machine (SVM) [121]. Thus, binding pockets and their druggability were extracted and analyzed, employing the default parameters.

#### 4.2.2. PockDrug

PockDrug predicts pocket druggability on both pockets guided and not guided by the ligand proximity [35] using different estimation methods based on 36 physicochemical and 16 geometrical descriptors to characterize each estimated pocket [122]. The prepared PDB file was uploaded to the PockDrug server (http://pockdrug.rpbs.univ-paris-diderot.fr/cgi-bin/index.py?page=Home, accessed on 25 July 2021) and assessed for binding sites regarding their 6 pocket descriptors (volume of convex hull (Å^3^); Kyte scale of residues’ hydrophobicity [123]; frequency of polar residues; frequency of aromatic residues; oxygen atoms’ frequency from Tyr residues in pockets [48]; and number of pocket residues) and corresponding average druggability probability, as well as its associated standard deviation. For a probability greater than 0.5, pockets are considered as druggable according to the published data [47]. Local pocket properties, sizes, shapes, and hydrophobicity were extracted for all the identified sites and annotated to pocket numbers.

#### 4.2.3. FTMap

The FTMap identifies binding hot spots of macromolecules (regions of the surface with major contributions to the ligand-binding free energy) using a computational mapping approach [54]. It uses small organic molecules as probes to sample the protein surface and scores them using the interaction energy [124]. Protein sites that bind different probes indicate this region as binding hot spots [54]. The prepared PDB file was uploaded to the FTMap server (https://ftmap.bu.edu/, accessed on 14 July 2021) and examined for the number of probes per cluster found according to the published protocols [64,125,126].

Similar to Borrel et al. [122], in this study, the predicted pocket of interest from the entire set of candidate cavities detected was selected as the one that best overlaps the crystallographic ligand-binding site and presented the best druggability index on all three programs.

#### 4.2.4. Molecular Docking on SARS-CoV-2 Mpro

Docking experiments were performed on previously prepared SARS-CoV-2 Mpro structures with the SeeSAR version 9.2 through sampling their spatial coordinates on the best interaction sites, which were defined by both the DoG site pocket finder [52,121] and druggability analysis from SeeSAR, PockDrug, and FTMap. Then, the docking library was loaded, including a set of NatProDB ligands previously filtered from QikPropTM and DerekTM, in addition to E64, which is a well know cysteine protease inhibitor, as a positive control. 

Docking calculations were done for each compound, generating 20 poses where each one has its binding affinity estimated from µM into the SARS-CoV-2 Mpro site. The best poses were selected based on their estimated affinity from the HYDE score function, while also considering the Torsion angle values to the binding conformation of the protein–ligand [61,121,127]. HYDE’s empirical score function (Equation (1)) relies on intrinsically balanced terms of atom-specific desolvation, hydration, and hydrogen bonds (H-bond; i.e., free of weighting parameters) based on the logP atomic increment system [128]. The binding affinity of a ligand, which is described as the quotient of ΔG and the non-hydrogen numbers of atoms in the compound (Equation (2)) [129], is expressed as follows.

ΔGHyde = Σatom i [ΔGi Dehydn + ΔGi h -bonds](1)

LE = ΔG⁄N(2)

where ΔG = -RT ln Ki and N = number of non-hydrogen atoms.

The selection of the best poses was based on their visual HYDE scores while also considering a statistics-based torsional analysis [127]. SeeSAR version 9.2 enables an interactive assessment of torsions and energies (in KJ·mol^−1^), including the desolvation (dehydration, −TΔS) and enthalpic (interaction, ΔH) contributions to the binding for both the protein and ligand. Furthermore, it also quantitatively reports the energy contributions for all heavy atoms (with a united atom approach for bounded H-atoms) and allows for a semi-quantitative estimation of the thermodynamic profile for all the tested compounds. Analyses, comparisons, and visualizations of the predicted binding pose predictions and all images were done by educational Pymol version 2.4.1 [55,130]. 

#### 4.2.5. SARS-CoV-2 Ligand Preparation

The Natural Products Database of the Bahia Semi-Arid region (NatProDB, http://natprodb.uefs.br/, accessed on 7 May 2021) contains 555 molecular structures publicly available [26]. All these molecules were automatically employed to perform an ADMET property analysis by QikProp^TM^ and Derek^TM^.

#### 4.2.6. ADMET Analysis by QikProp^TM^ and Derek^TM^

The predictions described below were made on a computer with 6 logical processors of an Intel^®^ Core TM i5-9400 and 4.10 GHz processor using the Windows 10 Professional operational system.

The QikProp program provides ranges to compare the physically significant descriptors and important ADMET properties of a specific compound with those of the 95% of approved drugs. Predicted human oral absorption on a 0 to 100% scale (HOA%) predicted apparent Caco-2 cell permeability in nm/sec (QPPCaco); predicted apparent Maden Darby Canine Kidney cell permeability (QPPMDCK); predicted the brain–blood partition coefficient (QPlogBB); and predicted the octanol–water partition coefficient (QPlogPo/w), among others. The program analyzes the chemical structure as fragments or as a whole and supports predictions about the structure in three dimensions. In addition, it flags 30 types of reactive functional groups that can cause false positives in high-throughput screening (HTS) assays [131,132]. 

Derek Nexus is an “expert rule-based” system for the prediction of toxicity [48,49,50] that uses a virtual base of molecules with reported toxicity alerts, including, for example, mutagenicity, genotoxicity, teratogenicity, carcinogenicity, and hepatotoxicity, for comparison with the potentially toxic group present in each query molecule [39]. The program generates an alert based on literature evidence and describes the potential toxicity for the complete structure [133].

#### 4.2.7. Molecular Dynamics (MD) Simulations on SARS-CoV-2 Mpro-NatProDB Complexes

The SARS-CoV-2 Mpro systems apo, holo, and five complexed with better-docked NatProDB compounds were submitted to molecular dynamic (MD) simulations using GROMACS 5.1.4 [71,72,73,74,75,76,134], available at the National Center for High-Performance Computing in São Paulo (CENAPAD-SP), employing the following parameters: 1 atm, 310 K, pH 7.4, GROMOS54A7 force field-updated [135], and PME [136] for electrostatic treatment with cut-off = 1.0 nm in a dodecahedral box solvated with water model SPC/E [137] with periodic boundary conditions (PBC). Na+ and Cl− ions were added to maintain the physiological salt concentration (0.15M) and to neutralize the residual system charge at pH = 7.4. At first, the system was energy-minimized (steepest descent/conjugate gradient) until forces reached ≤ 10 kJ·mol^−1^ and nm^−1^, followed by a pre-equilibrium simulation step (heavy atoms’ position restrained for 1 ns).

Finally, 100 ns of the unrestrained simulation was performed for all SARS-CoV-2 Mpro systems, where atomic coordinates were recorded every 10 ps for later analyses in an NPT ensemble with a V-rescale thermostat [138] and Berendsen barostat [139] with the SETTLE [140] algorithm for solvent bonds and the LINCS [141] algorithm for other bonds. The crystallographic ligand (RZS) and NatProDB compounds had their topology coordinates built in the Automated Topology Builder (ATB) version 3.0 server http://compbio.biosci.uq.edu.au/atb/, accessed on 9 May 2021 [142,143,144].

The 3D root-mean square fluctuation (RMSF) analysis converted RMSF data to B-factor from the RMSF module of GROMACS 5.1.4 [71,72,73,74,75,76,134]. The hydrogen bond analysis was conducted in the GROMACS H-bond module [77] and HbMap2Grace program [78], available on http://lmdm.biof.ufrj.br/software/hbmap2grace/index.html, accessed on 22 July 2021 and the molecular surface area (Å^2^; Table 1) analysis was conducted by the SurfinMD program [79], accessible on http://lmdm.biof.ufrj.br/software/surfinmd/index.html, accessed on 9 May 2021.

#### 4.2.8. Hydrogen Bond Capacity Analysis

Hydrogen bonds have been calculated between the number of donors/acceptors and the distance/angle between them, and there are few discussions about this relationship in drug discovery. Hydrogen bond capacity was quantitatively described [145,146] through an empirical definition. Wang et al. [56] described the hydrogen bond occurrence probability from MD simulation results and, based on their definition, we calculated the hydrogen bond capacity (Hbondcapac.) as defined by:

(3) Hbondcapac.=Hbond∑HBD,HBA

where <*Hbond*> is the average hydrogen bond number during the MD simulation, ∑*HBD*, while *HBA* is the sum of the hydrogen bond donor (HBD) and acceptor (HBA) of the molecule.

All steps performed in this study are described in Figure 1.

## 5. Conclusions

In this study, diverse and robust computer-aided drug discovery methods were sequentially employed against the main protease Mpro of SARS-CoV-2 (SARS-CoV-2 Mpro), aiming to select the most active molecules from the Natural Products Database of the Bahia Semi-Arid region (NatProDB, http://natprodb.uefs.br/, accessed on 7 May 2021). From the “top 10” docked compounds, some common scaffolds were selected (secotrachylobanoic acid, hexahydronaphthalen-1-yl, indolinedione, and benzopyran-4-one) indicating that Mpro could privilege the hydrophobic chains and (poly)aromatic rings. The best interactions with SARS-CoV-2 Mpro revealed that some enzyme sites were accessed, thereby confirming that this method can be employed as a suitable starting method for the identification of novel SARS-CoV-2 Mpro inhibitors.

The pharmacokinetic (ADMET) and toxicological analysis confirmed that nine out of the ten best molecules did not have any issues and could be employed in subsequent in vitro assays. Finally, this study should emphasize that the sequential application of in silico methods (ADMET analysis as well as docking and molecular dynamics) are valuable tools for searching the chemical space and selecting the best ligand structures that could be employed in experimental tests, minimizing the costs of testing many compounds. We also hope that the natural products from the Bahia Semi-arid region chosen here could be safe and effective for treating SARS-CoV-2 infection.

## Figures and Tables

**Table 1:** Best docking results carried out by SeeSAR version 9.2 (BioSolveIT GmbH) on the active site of the SARS-CoV-2 main protease (SARS-CoV-2 Mpro) using the Natural Products Database of the Bahia Semi-Arid region (NatProDB), E-64 (positive control), and RZS (crystallographic ligand, PDB ID: 5R82).

#	Compounds	Chemical Structure	Hydrogen Bond Donors (HBD) ^1^	Hydrogen Bond Acceptors (HBA)	Lipinki’s Rule ^2^ Violation	Molecular Surface Area (Å^2^) ^3^	Solvent Accessible Surface (Å^3^) ^4^	Estimated Affinity Range ^5^ (µM)	Inter Clash Type ^6^
b01	VE0DIA0AF	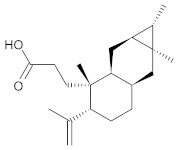	1	2	1	134.92	478.2	3.29–326.4	+
b02	VE0PPA0AF	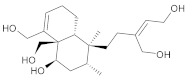	5	5	0	150.25	481.3	4.42–439.6	0
b03	VE0ISA0AF	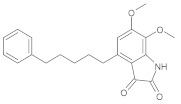	1	5	0	152.87	645.3	7.24–719.2	+
b04	VE0FKA0AF	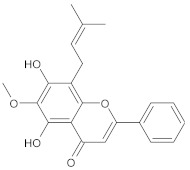	2	5	0	150.34	576.2	17.25–1714.3	+
b05	VE0FEA0SF	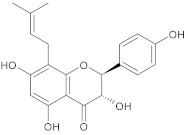	4	6	0	150.17	519.5	18.18–1806.5	0
b06	VE0JDA0SF	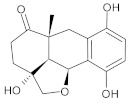	3	5	0	121.72	355.9	20.82–2068.7	0
b07	VE0ZDA0AF	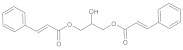	1	5	0	151.74	652.2	41.3–4103.0	+
b08	VE0NCA0SF	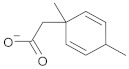	1	2	0	72.59	309.2	51.97–5164.2	+
b09	VE0KJA0SI	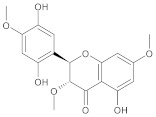	3	8	0	148.67	599.8	52.40–5206.5	0
b10	VE0NHA0SF	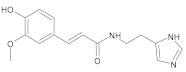	3	6	0	122.04	543.6	53.85–5350.2	+
255Control(RZS)	-	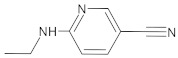	1	3	0	65.69	350.4	504.29–50,103.8	+
562Positive control(E-64)	-	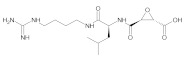	7	10	1	145.91	638.3	96,164.969, 455,201.9	0

^1^ The number of hydrogen bond donors (HBD), acceptors (HBA), and Lipinski’s rule violation were determined from the FAFF-Drugs server (https://fafdrugs4.rpbs.univ-paris-diderot.fr/, accessed on 21 April 2021) [56,57,58,59]. ^2^ Lipinski’s rule was described in [60]. ^3^ The molecular surface area were obtained from the pkCSM server (http://biosig.unimelb.edu.au/pkcsm/, accessed on 15 April 2021) [27]. ^4^ The solvent accessible surface (Å^3^) was calculated from the Marvin Sketch 18.8.0 Geometry plugin (http://www.chemaxon.com) employing the solvent radius 1.4 Å and pH = 7.4. ^5^ The estimated affinity (EA) ranges mark the lower and upper bound of the estimated affinities (±2 log-units). ^6^ Inter clash type: atomic clashes between the protein and docked ligand atoms as predicted by SeeSAR9.2. Inter clash type are classified as + (no clashes), 0 (few clashes), and − (many clashes).

## Data Availability

Not applicable.

## Supplementary Materials

The following are available online at https://www.mdpi.com/article/10.3390/ijms222111739/s1.

## Abbreviations

COVID-19	Coronavirus disease 2019
SARS-CoV-2	severe acute respiratory syndrome coronavirus 2
ACE2	angiotensin-converting enzyme
ADMET	absorption, distribution, metabolism, and excretion/toxicity
MD	molecular dynamics
NatProDB	Natural Products Database of the Bahia Semi-Arid region
3CLpro	SARS-CoV-2 Main Protease
CoVs	coronaviruses
PDB	Protein Data Bank
PSA	polar surface area
%HOA	human oral absorption in percentage
QPlogPo/w	logarithm of the partition coefficient in 1-octanol/water predicted by QikProp
QPLogBB	logarithm of the blood–brain barrier predicted by QikProp
QPPCaco	permeability across Caco-2 cells predicted by QikProp
CNS	central nervous system
RMSF	root-mean square fluctuation
LogKhsa	logarithmic human serum albumin-binding predicted by QikProp
EA	estimated affinities
Hbondcapac.	hydrogen bond capability
ΔEbinding	binding energy
QPPMDCK	permeability across Madin-Darby Canine Kidney cells predicted by QikProp
MW	molecular weight
HTS	high-throughput screening
