# Supplementary material for: Natural Products-Based Drug Design against SARS-CoV-2 Mpro 3CLpro
Source: Int J Mol Sci. 2021 Oct 29;22(21):11739. doi: 10.3390/ijms222111739 (PMC8583940; doi:10.3390/ijms222111739)
Supplement: Supplementary file 1 [file ijms-22-11739-s001.zip › ijms-1401045-supplementary.pdf]

Natural Products-based drug design against SARS-CoV-2 Mpro 3CLpro

Rai C. Silva[1,2*], Humberto F. Freitas[3,4], Joaquín M. Campos[5,6], Njogu M. Kimani[7], Carlos H. T. P. Silva[2], Rosivaldo S. Borges[1], Samuel S. R. Pita[4*], Cleydson B. R. Santos[1,8*]

[1] Graduate Program on Medicinal Chemistry and Molecular Modeling, Institute of Health science, Federal University of Pará. Augusto Corrêa, 01 - Guamá, Belém, 66075-110, PA, Brazil; raics@usp.br (R.C.S.); lqfmed@gmail.com (R.S.B);

[2] Departamento de Química, Faculdade de Filosofia, Ciências e Letras de Ribeirão Preto, Universidade de São Paulo, Ribeirão Preto 14040-901, SP, Brazil; tomich@fcfrp.usp.br (C.H.T.P.S.)

[3] Graduate Program on Pharmacy (PPGFAR-UFBA), Pharmacy College, Federal University of Bahia, Salvador, Bahia, Brazil; humbarato@gmail.com (H.F.F.);

[4] Laboratory of Bioinformatics and Molecular Modeling (LaBiMM), Federal University of Bahia, Av. Barão de Jeremoabo, 147, Pharmacy College, Ondina, Salvador, 40170-115, BA, Brazil; samuel.pita@ufba.br (S.S.R.P.)

[5] Department of Pharmaceutical and Organic Chemistry, Faculty of Pharmacy, Campus of Cartuja, University of Granada, 18071Granada, Spain; jmcampos@ugr.es (J.M.C)

[6] Biosanitary Institute of Granada (ibs.GRANADA), University of Granada, 18071-Granada, Spain.

[7] Department of Physical Sciences, University of Embu, P. O. BOX, Embu, 6-60100, Kenya; njogu.mark@embuni.ac.ke (N.M.K)

[8] Laboratory of Modeling and Computational Chemistry, Department of Biological and Health Sciences, Federal University of Amapá, 68902-280 Macapá, AP, Brazil; breno@unifap.br (C.B.R.S.)

* Corresponding author: raics@usp.br (R.C.S); breno@unifap.br (C.B.R.S); samuel.pita@ufba.br (S.S.R.P)

Supplementary Information

ORCIDS ID's:

RCS https://orcid.org/0000-0003-1774-4164; raics@usp.br

HFF https://orcid.org/0000-0003-3040-9694; humbarato@gmail.com

JMC https://orcid.org/0000-0002-9035-8123; jmcampos@ugr.es

NMK https://orcid.org/0000-0002-5171-1940;njogu.mark@embuni.ac.ke

CHTPS https://orcid.org/0000-0001-6049-3650; tomich@fcfrp.usp.br

RSB https://orcid.org/0000-0003-4072-7573;lqfmed@gmail.com

SSRP https://orcid.org/0000-0003-4053-8721; samuel.pita@ufba.br

CBRS https://orcid.org/0000-0002-0271-335X; breno@unifap.br

Figure S1: **RZS** (crystallographic ligand) docked on SARS-CoV-2 main protease calculated by SeeSAR. **Mpro** is shown as a cartoon (blue) with its main interacting residues (blue sticks), **RZS** is shown in magenta sticks and polar interactions are depicted as yellow dashed lines. This image was generated by educational pymol 2.4.1 [59].

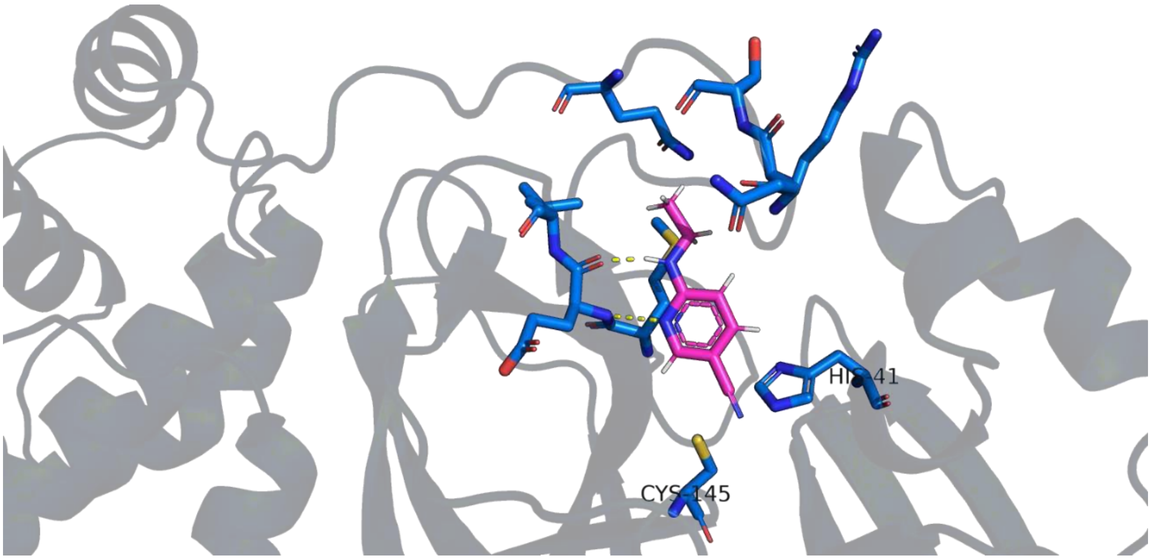

Figure S2: **E-64** (positive control) docked on SARS-CoV-2 Mpro calculated by SeeSAR. **Mpro** are shown as cartoon (blue) with its main interacting residues (blue sticks), **E-64** is shown in orange sticks and polar interactions are depicted as yellow dashed lines. This image was generated by educational pymol 2.4.1 [59].

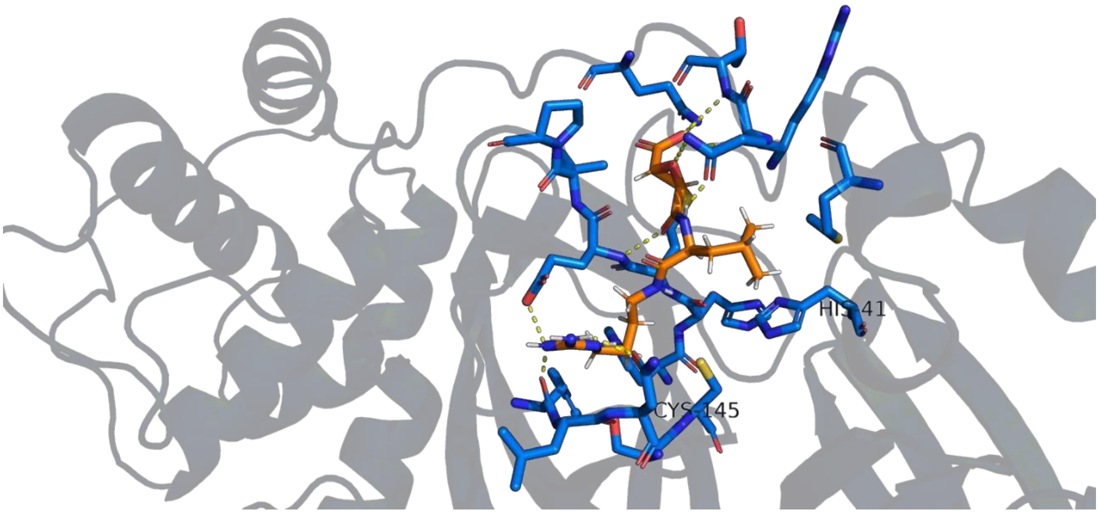

Figure S3: Better energy ranked NatProDB compound **b01** docked on SARS-CoV-2 main protease calculated by SeeSAR. **Mpro** are shown as cartoon (blue) with its main interacting residues (blue sticks), **b01** is shown in green sticks and polar interaction is depicted as yellow dashed lines. This image was generated by educational pymol 2.4.1 [59].

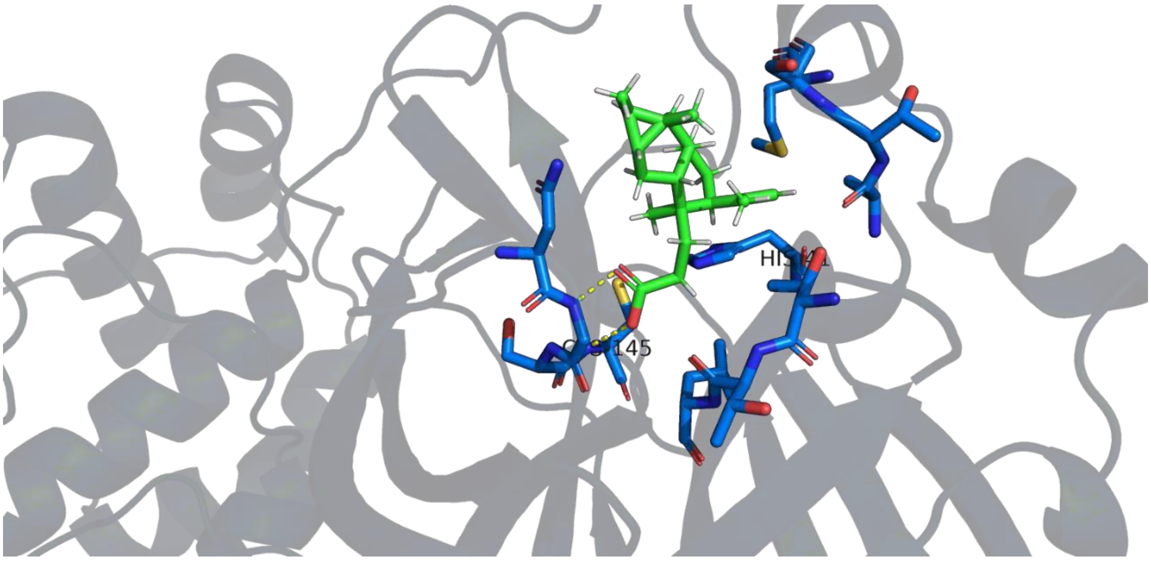

Figure S4: Second-best energy ranked of NatProDB compound **b02** docked on SARS-COV-2 Mpro calculated by seeSAR. **Mpro** are shown as cartoon (blue) with its main interacting residues (blue sticks), **b01** is shown in cyan sticks and polar interaction is depicted as yellow dashed lines. this image was generated by educational pymol 2.4.1 [59].

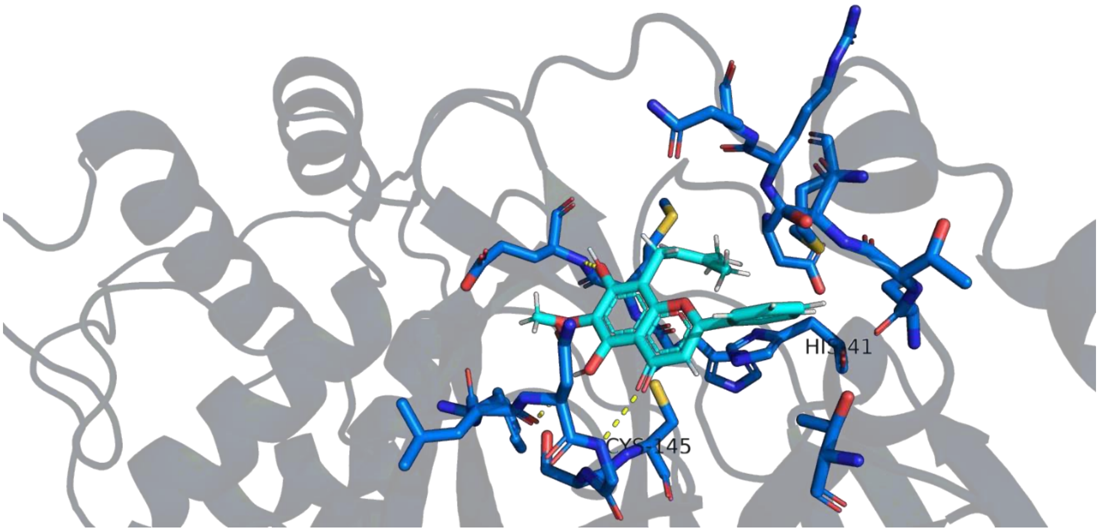

Figure S5: Molecular dynamics (MD) simulation data for main protease of SARS-CoV-2 main protease complexes generated for gromacs 5.1.4 [66- 72]. a) rmsd values (averaged mean ± standard deviation) for each system: **apo** (0.31 ± 0.03), **holo** (0.44 ± 0.04), **E-64** (0.31 ± 0.03), **b01** (0.33 ± 0.03), **b02** (0.35 ± 0.03), **b03** (0.38 ± 0.03), **b04** (0.43 ± 0.03), **b05** (0.37 ± 0.03); b) rmsf and c) radius of gyration, both from productive phase (40-100ns).

A)

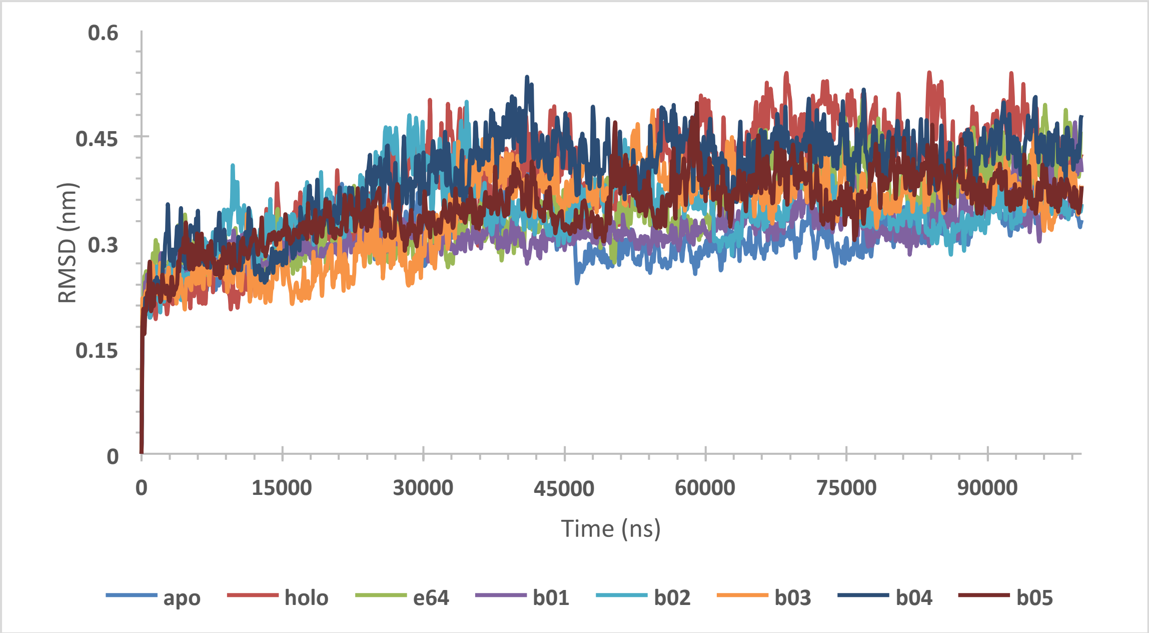

B)

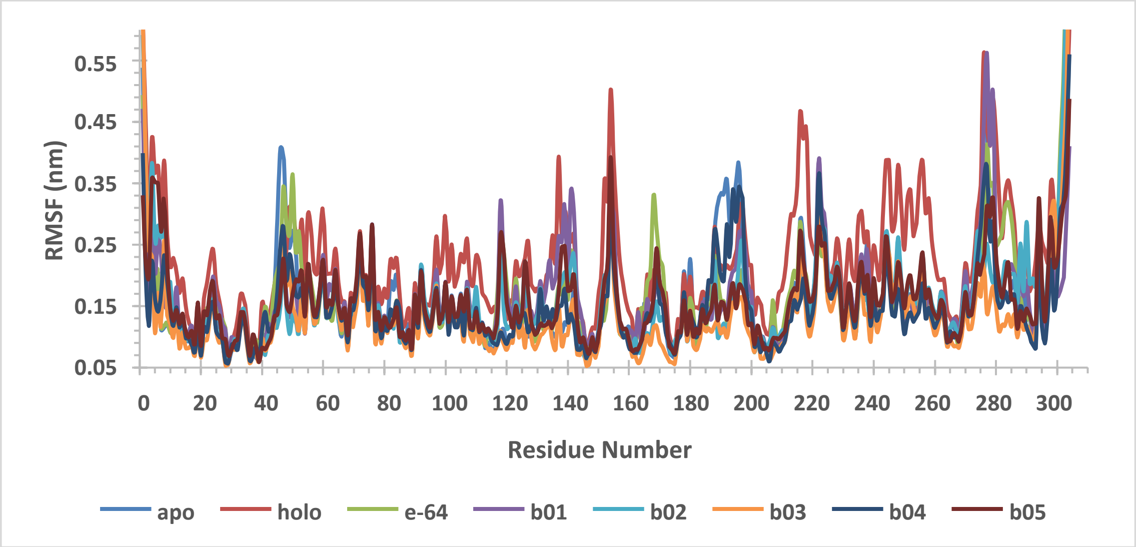

C)

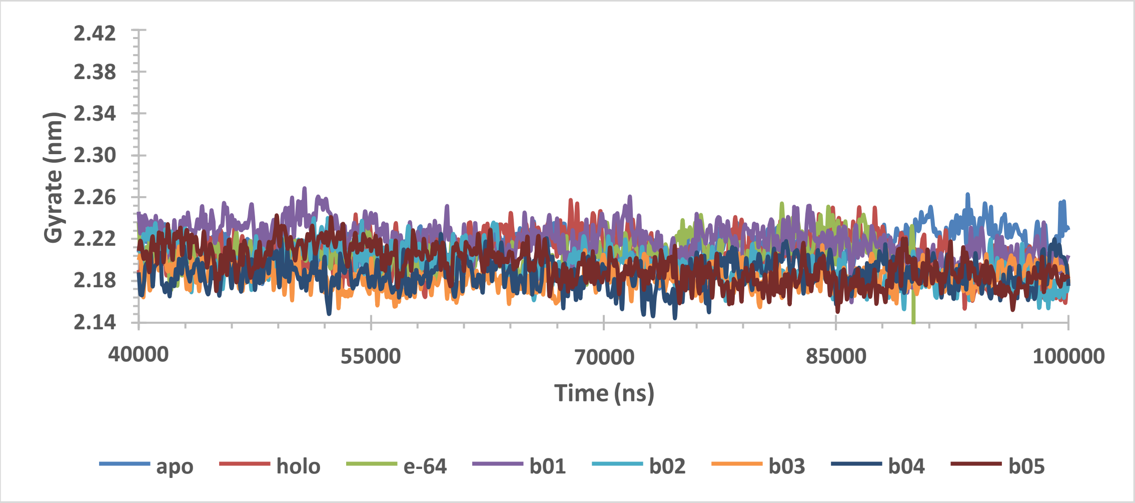

Figure S6: Productive phase (40-100ns) Secondary structure evaluation of SARS-CoV- 2 Main protease complexes by DSSP 3.1.4 [63-65] module installed on GROMACS 5.1.4 [66-72]. From upper left corner to the right: apo, holo, **E-64**, b01, b02, b03, b04, b05.

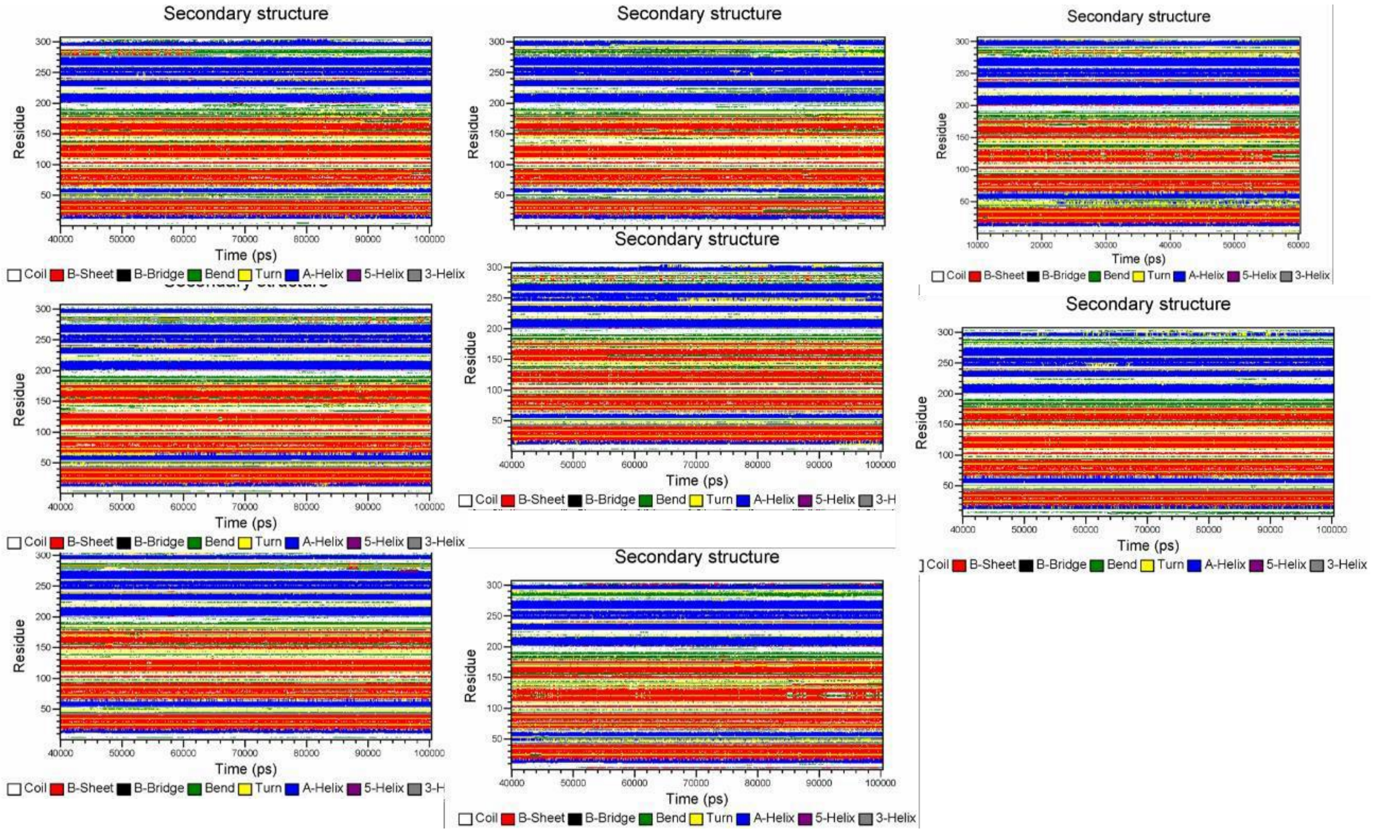

Table S1: ADMET properties calculated from QikProp™ for NatProDB compounds on SARS-CoV-2 Main protease structure.

Nº	SMILES code	#stars [a]	CNS [b]	HOA [c]	Caco-2 [d]	log P [e]	PSA (Å²) [f]	HBD [g]	HBA [h]	RO5 [i]	logKHSA [j]	QPlogHERG [k]
**b01**	C=C(C)C3CCC2CC1(C)C(C)C1CC2C3(C)CC C(=O)O	1	-1	87.94	297.61	6.69	49.54	1	2	1	0.84	-1.92
**b02**	CC2CC(O)C1(CO)C(C O)=CCCC1C2(C)CC/C (=C/CO)CO	0	-2	100.0	759.23	4.52	81.69	5	5	0	0.18	-5.14
**b03**	COc3cc(CCCCCc1cccc c1)c2c(=O)c(=O)[nH]c2 c3OC	0	-2	64.56	304.20	0.15	70.26	1	5	0	-0.36	-3.47
**b04**	COc3c(O)c(C/C=C(C)\ C)c2oc(c1ccccc1)cc(=O) c2c3O	0	-1	100.0	1101.54	5.03	69.21	2	5	0	0.65	-5.53
**b05**	C/C(C)=C\Cc2c(O)cc(O)c3C(=O)C(O)C(c1cc c(O)cc1)Oc23	0	-1	77.03	320.64	0.27	107.35	4	6	0	-0.29	-3.20
**b06**	CC24Cc1c(O)ccc(O)c1 C3OCC(O)(CCC2=O) C34	0	-2	96.23	404.10	3.91	92.32	3	5	0	0.29	-6.48
**b07**	O=C(/C=C/c1ccccc1)O CC(O)COC(=O)/C=C/c 2ccccc2	0	-2	75.28	108.69	3.32	90.45	1	5	0	0.08	-4.53
**b08**	CC1C=CC(C)(CC(=O) O)C=C1	0	-1	85.35	347.02	2.53	46,80	1	2	0	-0.28	-1.56
**b09**	COc3cc(O)c2C(=O)C(OC)C(c1cc(O)c(OC)cc 1O)Oc2c3	0	-2	80,30	256,82	0.68	95.75	3	6	0	-0.28	-5.77
	**E-64**	1	-2	14.41	1.52	-3.93	46.80	7	10	1	-1.31	0.16

[a] Number of computed properties which fall outside the required range for 95% of known drug: 0 to 5;

[b] Central Nervous System Activity: -2 (inactive) to +2 (active);

[c] Human Oral Absorption (%): HOA >80% high and <25% poor;

[d] Apparent Permeability on Caco-2 cell membrane (Boehringer-Ingelheim scale for 95% of the drugs): <5 nm /s low and >500 nm /s, high;

[e] Logarithmic Partition Coefficient (*n*-octanol and water phases): -2 to 6.5;

[f] Van der Waals Surface Area of polar nitrogen and oxygen atoms: 7.0 to 200.0 Å²;

[g] Hydrogen bond Donor (number): 0 to 6;

[h] Hydrogen bond Acceptor (number): 2 to 20;

[I] Violations of Lipinski's 'Rule of Five' (RO5);

[j] Logarithmic Human Serum Albumin Binding: -1.5 to 1.5;

[k] Predicted IC_{50} value for HERG K+ channels blockage: below –5.

Table S2: Noncovalent interaction predictions for better SARS-CoV-2 Main protease complexescalculated from Protein-Ligand Interaction Profiler.

PLIP Interactions							
**Compounds**	**Hydrophobic Interactions**	**Distance (Å)**	**Hydrogen Bonds**	**Distance Donor-Acceptor (Å)**	**Angle (°)**	**Salt Bridges**	**Distance (Å)**
**(b01)**	Leu27	3.84	Gly143	2.78	166.93		
**(b02)**	Asn142	3.71	**Cys145**	2.86	134.14		
			Phe140	2.78	145.72		
			His163	2.8	142.21		
			Met165	4.04	145.02		
			Glu166	2.95	166.16		
**RZS**	-	-	**His41**	3.94	122.08		
			Glu166	2.94	170.4		
**E-64**	Met49	3.69	Asn142	3.93	118.92	Glu166	4.37
			Asn142	2.91	131.69	Glu166	3.99
			Asn142	2.71	143.03		
			Glu166	2.85	143.18		
			Arg188	4.08	147.4		
			Gln189	2.67	144.09		
			Gln189	3.29	100.56		
			Thr190	3.34	147.49		
			Gln192	2.83	127.77		

Table S3: Molecular Surface Area and Hydrogen bond interactions with SARS-CoV-2 Main protease (**Mpro**) active site residues and best docked ligands from Natural Products Database of Bahia Semi-Arid region (NatProDB), **E-64** (positive control) and **RZS** (crystallographic ligand, PDB ID: 5R82).

Mpro subsites	holo Residue	holo Number	holo Area (A2)	**E-64** Residue	**E-64** Number	**E-64** Area (A2)	b01 Residue	b01 Number	b01 Area (A2)	b02 Residue	b02 Number	b02 Area (A2)	b03 Residue	b03 Number	b03 Area (A2)	b04 Residue	b04 Number
	PHE	140	20.3	**HIS**	**41**	**27.0**	**HIS**	**41**	**19.5**	**HIS**	**41**	**22.1**	**HIS**	**41**	**22.9**	**HIS**	**41**
	SER	144	5.2	GLY	143	13.5	SER	144	7.4	GLY	143	13.8	ASN	142	25.4	LEU	141
	HIS	163	13.7	SER	144	5.7	**CYS**	**145**	**17.6**	**CYS**	**145**	**23.8**	GLY	143	19.3	ASN	142
S1				**CYS**	**145**	**21.0**	GLU	166	8.9	GLU	166	19.3	SER	144	5.0	GLY	143
				GLU	166	21.2							**CYS**	**145**	**16.3**	SER	144
													HIS	164	10.4	**CYS**	**145**
																GLU	166
	-	-	-	MET	49	6.7	MET	49	15.2	MET	49	24.5	ASP	48	9.3	MET	49
	-	-	-	MET	165	14.6	MET	165	13.1	MET	165	6.2	MET	49	7.8	MET	165
	-	-	-	GLN	189	13.0	GLN	189	9.1	GLN	189	13.1	MET	165	9.0	GLN	189
S2													ASP	187	6.7		
													ARG	188	5.7		
													GLN	189	22.5		
	-	-	-	THR	25	21.8	THR	25	18.3	THR	25	13.8	THR	25	16.0	THR	25
S1'	-	-	-	THR	26	21.0	THR	26	11.7	THR	26	16.4	THR	26	20.0	LEU	27
	-	-	-	LEU	27	17.9	LEU	27	15.6	LEU	27	16.5	LEU	27	16.8	-	-
	-	-	-	CYS	44	7.0	VAL	42	12.0	SER	46	11.6	CYS	44	15.6	CYS	44
	-	-	-	SER	46	14.2	CYS	44	18.6	TYR	118	6.3	THR	45	6.7	SER	46
New	-	-	-	ASN	119	9.1	THR	45	11.4	ASN	119	12.9	SER	46	19.7	LEU	50
							SER	46	17.6				ASN	119	23.0		

Mpro subsites	holo Donor	holo Acceptor	holo Time Simulation (%)	**E-64** Donor	**E-64** Acceptor	**E-64** Time Simulation (%)	b01 Donor	b01 Acceptor	b01 Time Simulation (%)	b02 Donor	b02 Acceptor	b02 Time Simulation (%)	b03 Donor	b03 Acceptor	b03 Time Simulation (%)	b04 Donor	b04 Acceptor	b04 Time Simulation (%)	b05 Donor	b05 Acceptor	b05 Time Simulation (%)
				**H41**	**O1**	**10.18**	S144	O2	85.19	**H41**	O4	38.6	G143	O4	85.36	N142	O3	5.82	S144	O17	25.46
				**H41**	**O5**	**9.98**	S144	O1	84.69	E166	O4	34.94	G143	O3	47.25	G143	O4	63.39	E166	O19	7.32
				E166	O5	43.31	C145	O2	60.73	O4	E166	7.32	S144	O3	59.4	S144	O4	75.87	Q189	O20	89.85
S1				N3	E166 OE1	29.94	C145	O1	60.23				**C145**	**O3**	**72.21**	S144	O5	90.85	Q189	O19	42.93
				N3	E166 OE2	75.05							N1	H164	97	**C145**	**O4**	**93.51**	O26	HIS41 O	71.38
				N5	G143	8.18										O2	E166 OE1	47.75	O17	SER144O	5.32
				N5	E166 OE1	5.79										O2	E166 OE2	53.24	O18	GLU166OE1	25.62

				N5	E166 OE2	29.54										O5	L141	14.31	O18	GLU166OE2	74.88
S2				N1	D187	5.39				Q189	O5	11.48									
										O5	Q189	45.26									
				N4	THR 26O	24.75				T25	O2	5.82							T25	O26	61.4
S1'				N5	THR 26O	26.35				T26	O1	12.65									
										T26	O1	18.97									
										O1	T26	11.31									
	Y118	N2	15.1	T190	O4	68.46				Y118	O2	7.65							S46	O20	47.75
	N1	S121	6.5	T190	O3	62.08				Y118	O1	6.32							O26	C44	98.17
				A191	O4	72.85				N119	O1	32.11									
				A191	O3	70.26				O1	N119	10.82									
New				Q192 N	O4	59.28															
				Q192 N	O3	61.08															
				Q192 NE2	O4	50.3															
				Q192 NE2	O3	45.51															
				N4	N119	5.79															
				N5	N119	20.76															
Hbnum		0.5			8.4			3.2			3.2			3.8			6.3			5.8	
Hbond$_{capac.}$ [1]		0.13			0.49			1.06			0.32			0.6			0.9			0.6	
Active Site Volume (Å^3)[2]		430.6			461.8			349.6			303.9			329.0			262.9			319.7	
ΔE$_{binding}$ MM-PBSA[3]		-			-17.65 ± 0.07			-12.51 ± 0.05			-15.10 ± 0.04			-19.15 ± 0.04			-17.59 ± 0.14			-16.35 ± 0.04	

[1] See Materials and Methods for definition

[2] The active site volume was calculated from Fpocket module [77, 78] from MD productive phase.

[3] The MM-PBSA energies were calculated from *g_mmpbsa* program [80, 81].
